# VISION LOSS AND FOOD INSECURITY IN THE NATIONAL HEALTH AND AGING TRENDS STUDY

**DOI:** 10.1093/geroni/igad104.0352

**Published:** 2023-12-21

**Authors:** Louay Almidani, Moon Lee, Bonnielin Swenor, Laura Samuel, Varshini Varadaraj

**Affiliations:** Wilmer Eye Institute, Baltimore, Maryland, United States; Wilmer Eye Institute, Baltimore, Maryland, United States; Johns Hopkins University, Baltimore, Maryland, United States; Johns Hopkins University School of Nursing, Baltimore, Maryland, United States; Johns Hopkins University, Baltimore, Maryland, United States

## Abstract

Adults with vision impairment (VI) may be at a greater risk of experiencing food insecurity due to economic hardships and difficulty with daily activities. However, the relationship is not well understood. We examined the cross-sectional associations between VI and food insecurity in older community-dwelling US adults among 2,815 National Health and Aging Trends Study participants. VI was measured on categorical (distance acuity VI [> 20/40], near acuity VI [> 20/40], contrast sensitivity impairment [CSI] [<1.55], and any VI (distance, near, or CSI)) and continuous scales (distance and near acuity [per 0.1 logMAR], and contrast sensitivity [per 0.1 logCS units]). Food insecurity was classified based on functional limitations, lack of social support, and/or financial limitations. Overall, older adults with any VI had higher prevalence rates of food insecurity than adults without VI (4.56% vs. 1.90%). In fully-adjusted models, older adults with any VI had double the odds of food insecurity than their peers (OR=2.13, 95% CI: 1.25–3.62). Although no associations were found when assessing other vision measures categorically, continuous scales showed strong associations; worse distance (OR=5.00, 95% CI: 1.85–13.48) and near acuity (OR=2.56, 95% CI: 1.05–6.21, per 0.1 logMAR), and worse CS (OR=2.57, 95% CI: 1.11–5.94, per 0.1 logCS units) were associated with higher odds of food insecurity. These results suggest that older Americans with VI have a higher likelihood of food insecurity, and highlight a need to examine strategies, such as more disability-inclusive policies impacting food access, to improve accessibility and reduce these inequities.

